# Nature-based mindfulness program for parents and caregivers: a mixed-methods study

**DOI:** 10.3389/fpsyg.2026.1900785

**Published:** 2026-10-15

**Authors:** Zoe Jiwen Zhang, Herman Hay Ming Lo, Wai Fong Lok, Qi-rong Chen

**Affiliations:** 1 Kunming Medical University, Kunming, China; 2 Department of Applied Social Sciences, The Hong Kong Polytechnic University, Hong Kong, Hong Kong SAR, China; 3 Hong Kong Family Welfare Society, Hong Kong, Hong Kong SAR, China

**Keywords:** caregivers, Chinese, mindfulness-based program, nature, parents

## Abstract

**Introduction:**

There is a growing interest in incorporating nature into mindfulness-based programs. This study is unique as it targets parents and family caregivers experiencing caregiving stress.

**Methods:**

A mixed-methods study was conducted to evaluate the outcome of a nature-based mindfulness intervention program. A comprehensive outcome measures targeting benefits of parents and caregivers, including mental health symptoms, wellbeing, physical distress, family functioning, and family conflicts, were assessed over time. The role of nature in overall outcome was investigated through the participants' perceived connectedness to nature and time spent in nature. The program consists of four sessions, totaling 8 h of contact time, held at a retreat center and social care units. A total of 95 participants were recruited, including 40 caregivers of older adults and 55 parents under stress. The study employed a time series design, with assessments conducted 1 month before the program, pre-program, post-program, and at a 3-month follow-up.

**Results:**

For caregivers, significant improvements were observed in multiple outcome measures, including anxiety, stress, physical distress, daily functioning, and wellbeing, following the program. Further improvement in connectedness with nature was noted between the pre-test and the 3-month follow-up, with depression and family functioning approaching significance. For parents under stress, significant improvements were found only in wellbeing after the program. However, additional improvements in anxiety, stress, physical distress, and family conflicts (psychological aggression, corporal punishment, and physical maltreatment) were observed during the follow-up period, with depression and daily functioning approaching significance. Hierarchical regression analysis indicated that connectedness to nature significantly associated with the program's effect on depression. Qualitative analysis revealed that participants benefited from attention restoration, increased awareness, and insights gained from nature and mindfulness practices.

**Discussion:**

Due to the limitations of the study, including the lack of control group and the use of self-reported measures, future study should verify the benefits of nature-based mindfulness programs particularly on the effects on parents and caregivers under stress and at risk of mental health conditions.

## Introduction

Mindfulness originates from Eastern traditions and refers to the practice of maintaining awareness and directing attention to the present moment in purpose (Kabat-Zinn, 2013). The Mindfulness-Based Stress Reduction (MBSR) program was introduced at the University of Massachusetts Medical School in 1979 (Kabat-Zinn, 2013), and Mindfulness-Based Cognitive Therapy (MBCT) was developed as a new form of cognitive behavioral therapy to prevent relapses in major depression (Kuyken et al., 2016; Segal et al., 2012) and many other mental health conditions in health care contexts (Demarzo et al., 2015). Overall, mindfulness-based programs (MBPs) like MBSR and MBCT aim to provide a scientifically grounded, educationally informed approach to managing mental and physical wellbeing and promote overall health. Various adaptations have led to the formulation of diverse curricula for MBPs (Crane, 2017).

There is growing attention to incorporating nature into mindfulness-based programs (Djernis et al., 2019). Nature can be defined as “areas containing elements of living systems that include plants and non-human animals across a range of scales and degrees of human management, from a small urban park through to relatively ‘pristine wilderness”' (Bratman et al., 2012; Scopelliti et al., 2019). Nature is regarded as an essential therapeutic component that can lead to positive outcomes in physical health, mental health, and wellbeing (Jimenez et al., 2021; Tester-Jones et al., 2020). Spending time in nature promotes immune functioning, decreases the risk of cardiovascular disease, improves interpersonal functioning, and reduces stress and emotional distress (De Vries et al., 2013; Kotera et al., 2022; Kuo, 2015; Lackey et al., 2021).

MBPs originated from traditional spiritual practices across religions, often taking place in remote and rural areas. Such geographical locations provide opportunities for restoration, transformation, and personal reflection (Farb, 2014; Gill et al., 2019; Surinrut et al., 2016). Engaging in an extended meditation retreat can lead to benefits by allowing individuals to focus on their spiritual practice, temporarily disconnect from daily stressors, and immerse themselves in a unique environment (Khoury et al., 2016). In contemporary societies, MBPs are often organized in secular contexts such as hospitals, clinics, and other social care service units (Kabat-Zinn, 2003). These programs are typically offered in a brief format and require participants to travel to attend. The advantage of organizing MBPs in these venues is their proximity and convenience in terms of geographical location. One potential direction for advancing and innovating MBPs is to integrate nature into the programs to maximize mental health and wellbeing outcomes (Burger et al., 2024; Lymeus et al., 2019). Careful program planning is required to ensure the potential benefits of a natural setting, as transportation logistics can become burdensome for participants and organizers.

Learning and practicing mindfulness require regular practice to observe one's experiences, starting from internal physical sensations, feelings, and thoughts. It is easier for meditators to tune into a “being” mode for meditation, which facilitates attention regulation for managing stress and challenges. A study supported the idea that arranging a mindfulness program in nature reduced dropout rates and improved homework completion, suggesting that nature-based mindfulness programs can maximize the benefits of the program (Burger et al., 2024; Vecchio et al., 2026). While several studies on the outcomes of nature-based mindfulness programs have been published in the past decade, most have focused on university students (Choe et al., 2020; Lymeus et al., 2018; Nisbet et al., 2019). Spending time in nature promotes health, prevents mental health problems such as stress, depression and anxiety, and improves interpersonal functioning (Choe et al., 2020; Lymeus et al., 2018; Nisbet et al., 2019; Owens and Bunce, 2023). Attention restoration theory is a key framework that explains the benefits of nature and its application to nature-based mindfulness programs. According to this theory, exposure to nature supports the restorative process. Natural settings are often physically distant from one's stressful daily life, allowing for effortless attention to be drawn to fascinating objects (Kaplan, 2001). The process of attention restoration can be divided into three aspects: (1) Being away and soft fascination: Nature allows individuals to distance themselves from their day-to-day environment and mental activity, promoting both physical and conceptual transformations. A different environment can help change perspectives and facilitate the necessary experiential shifts for change. Nature offers a stream of stimuli, such as trees, clouds, sunsets, and rivers, that are both fascinating and different from the usual. It engages the mind and helps individuals see, experience, and feel, capturing attention, restoring focus and mental capacity, and reducing fatigue. (2) Extent: Nature provides a new environment that is rich and coherent enough to form a sense of relatedness, where one's perceptions are part of a larger whole. (3) Compatibility: The new environment is compatible with the purposes and intentions of MBPs and the participants themselves. It aligns with what individuals are trying to do and achieve. Many participants face challenges in practicing mindfulness as they are occupied with thoughts and emotions. A natural environment provides support, making it easier for participants to shift their attention to mindfulness exercises with self-care and self-compassion.

Many models of parenting are rooted in social learning theory (Bornstein, 2002). Numerous support and education programs for parents and caregivers have focused on enhancing knowledge and skills, utilizing universal principles of behavioral approaches to improve behavior management and address related problems (Sanders and Morawska, 2018; Schulz et al., 2020). In contrast, MBPs for parents and caregivers emphasize cultivating a mindful and conscious relationship between parents, caregivers, and care recipients (Kabat-Zinn and Kabat-Zinn, 2021), aiming to foster awareness and highlight the value of providing quality attention and unconditional care. Instructors of MBPs for parents and caregivers do not offer specific behavioral strategies. Instead, participants are supported to observe their own limits and to bring awareness and acceptance to themselves, responding to their family members or children with conscious choices rather than reacting emotionally (Bögels and Restifo, 2013).

MBPs should be culturally sensitive and tailored to the specific needs of the population they serve. Studies have reported that parents of children with special needs and family caregivers have worse health profiles, more medical consultations, anxiety and depression, weight loss, and lower quality of life (Ho et al., 2020; Ren et al., 2020). It is more alarming that abusive behaviors toward children and older adults predicted by the anxiety and depression of caregivers, as such associations are mediated by burnout, extended hours of care, and caregiving burden (Cooper et al., 2010; Mikolajczak et al., 2018). In the context of East Asian societies, where filial piety is highly valued, it is crucial to address the cultural expectations and challenges faced by adult children caring for their aging parents (Chan et al., 2012). Difficulties encountered by parents and family caregivers are often compounded by a lack of accessible and feasible community care services, and this disconnect between ideal expectations and performance feasibility can result in feelings of shame and guilt, as well as blame from family and community (Canda, 2013). MBPs for parents and caregivers can provide valuable support and coping strategies. These programs empower caregivers to manage their stress, enhance their wellbeing, and improve their caregiving experience (Lo et al., 2020; Zhang et al., 2025).

The objectives of this study are: (1) To examine the mental health outcomes (depression, anxiety, and stress) of a nature-based mindfulness intervention program for parents and caregivers who have experienced high levels of caregiving stress. (2) To examine the effects of the program on enhancing caregivers' psychosocial functioning and coping (physical distress, daily functioning, wellbeing, self-compassion, and connectedness with nature). (3) To explore the role of nature in promoting the outcome of MBPs.

Hypotheses of the study include: (1) Parents and caregivers did not report significant changes from T0 to T1 before the nature-based MBP started. It served to establish the stability of the outcome measures before the program commenced, allowing the research team to assess whether the changes observed after T1 were likely to be effect of the program. (2) Parents and caregivers reported significant improvements in mental health outcomes (depression, anxiety, and stress) and other psychosocial functioning outcomes (physical distress, daily functioning, wellbeing, family functioning, psychological abuse, physical abuse, psychological aggression, corporal punishment, self-compassion, and connectedness to nature) from T1 to T2 during the implementation of the nature-based MBP. (3) Parents and caregivers reported significant improvements in mental health outcomes (depression, anxiety, and stress) and other psychosocial functioning outcomes (physical distress, daily functioning, wellbeing, family functioning, psychological abuse, physical abuse, psychological aggression, corporal punishment, self-compassion, and connectedness to nature) from T2 to T3 during the follow-up period after the nature-based MBP. (4) Connectedness to nature, as perceived by parents and caregivers, and time spent in nature associated with improvements in outcomes.

## Methods

An explanatory sequential mixed-methods study was conducted to address the research objectives (Thyer, 2023). A total of four consecutive time points were assessed before and after the intervention, as the design of a randomized controlled trial was considered as not suitable due to the limitations in service operation (Shadish et al., 2002). The most significant improvements were expected to emerge between T1 (right before the intervention) and T2 (immediately after the intervention). The quantitative component aimed to investigate the outcomes of the nature-based mindfulness program and the role of nature in enhancing these outcomes. MutThe qualitative component builds on the results and attention restoration theory to explain the results using participants' subjective experiences and their perceived benefits from the program.

### Quantitative study

#### Procedures

An NGO in Hong Kong operates a family retreat center located in the rural area of the city (see https://www.youtube.com/watch?v=OT_x-cN0-ZY). The nature-based MBP program was promoted among family and elderly service units, as well as kindergartens. Social workers from the NGO and partner organizations recruited parents and family caregivers to participate in this project.

This study employed a time-series design (Thyer, 2023). To investigate the outcomes and their maintenance of the program, all participants were invited to complete the same set of questionnaires at four time points: 1 month before the program (T0), pre-program (T1), post-program (T2), and 3-month follow-up (T3). All selected scales have been validated in Chinese samples. Completing the questionnaires took less than 30 min. This study was approved by the Ethics Committee of the University (HSEARS20220919003). The informed consent of all participants was obtained. This study was conducted under the principles of the Declaration of Helsinki.

#### Intervention

The nature-based MBP is an adapted version of a low-intensity MBP that has been validated with Chinese parents and caregivers (Lo et al., 2017). Within a fast-paced and high-pressure living conditions in Hong Kong, a low-intensity intervention design was selected to address the challenges in relation to caregivers' household tasks and work-family balance. Many parents and caregivers expressed their preferences to a brief support program, instead of an intensive MBP lasting 20 h or more. The program included brief mindfulness practices (body scan, mindful movement, and mindful sitting) lasting 15 to 20 mins, and homework audio guidance at similar duration were provided for participants. In addition to mindfulness practices, qualified instructors addressed inquiries in mindfulness exercises, and provided psychoeducation on stress reactivity, and strategies of self-care. The original program was developed for parents of children with developmental challenges, and it was later adapted to apply in parents from disadvantaged families (Lo et al., 2019), caregivers of adults with early psychosis (Zhang et al., 2024), and frail older adults (Zhang et al., 2025). Results of these studies have shown that low-intensity MBPs can significantly reduce parenting stress, improve coping, increase positive caregiving experiences, among parents and caregivers. The program protocol was similar to the trial for caregivers of frail older adults (Lo et al., 2022), and the major differences between two was to organize some of the sessions in this study in the family retreat center in the rural area.

The Nature-based MBP consists of four sessions totaling 8 h of contact time, including sessions held in ordinary social care service units and at the family retreat center. The time spent at the retreat house and ordinary social care service units was jointly determined by the project worker and the social worker, allowing for flexibility. In total, 10 groups were organized. For sessions organized at the family retreat center, the NGO provided free transportation for parents and caregivers to travel from their district to the center. The family retreat center offers a tranquil, home-like environment and facilities ideal for MBPs. Its geographical location allows participants to travel to the venue within an hour from the city and enjoy the benefits of experiencing connectedness to nature. Table 1 summarizes the themes and mindfulness practices in each session. Instructors received relevant MBP professional training and have over 10 years of experience in teaching MBPs.

**Table 1:** The session plans of nature-based mindfulness program.

Themes	Mindfulness practice	Possible nature-based activities
**Session 1** Stepping out of automatic mode	Mindful eating body scan	Mindfulness of five senses
**Session 2** Reacting vs. responding	3-min breathing mindfulness of breathing	Mindful stretching
**Session 3** Creating an inner space	Mindfulness of body and breath	Mindful walking mountain meditation
**Session 4** Bringing compassion to life	Body scan; Mindful sitting; Befriending exercise	

#### Participants

A total of 103 parents and caregivers expressed interest in participating in the program. However, 8 did not attend any sessions or complete the study questionnaires. The research team received 95 sets of questionnaires at T0 and T1. Among these participants, 40 were caregivers of older adults, and 55 were parents of children. The majority of participants were female (91.6%) and married (73.7%), and they were primarily responsible for the care recipients. Approximately half of the caregivers were caring for an older adult with dementia, while about half of the parents had a child with ADHD. This indicates that the participants were facing significant challenges in their mental health conditions. Table 2 summarizes the participants' profiles, and Figure 1 illustrates the participant flow in this study.

**Table 2:** Participant profile.

	Caregivers (*n* = 40)	Parents (*n* = 55)	Total (*n* = 95)
Gender			
Male	5 (12.5%)	3 (5.5%)	8 (8.4%)
Female	35 (87.5%)	52 (94.5%)	87 (91.6%)
Age			
Below 40	0 (0.0%)	19 (34.5%)	19 (20.0%)
40–49	2 (5.0%)	26 (47.3%)	28 (29.5%)
50–59	5 (12.5%)	7 (12.7%)	12 (12.6%)
60–69	14 (35.0%)	3 (5.5%)	17 (17.9%)
Over 70	19 (47.5%)	0 (0.0%)	19 (20.0%)
Education			
Below primary	2 (5.0%)	0 (0.0%)	2 (2.1%)
Primary	5 (12.5%)	9 (16.4%)	14 (14.7%)
Secondary	14 (35.0%)	37 (67.3%)	51 (53.7%)
Tertiary	19 (47.5%)	8 (14.5%)	27 (28.4%)
Missing	0 (0.0%)	1 (1.8%)	1 (1.1%)
Marital status			
Single	8 (20.0%)	1 (1.8%)	9 (9.5%)
Married	28 (70.0%)	42 (76.4%)	70 (73.7%)
Divorced/Separated	1 (2.5%)	10 (18.2%)	11 (11.6%)
Widowed	3 (7.5%)	1 (1.8%)	4 (4.2%)
Missing	0 (0.0%)	1 (1.8%)	1 (1.1%)
Work status			
Unemployed	4 (10.0%)	39 (70.9%)	43 (45.3%)
Retired	32 (80.0%)	5 (9.1%)	37 (38.9%)
Part-time	3 (7.5%)	7 (12.7%)	10 (10.5%)
Full-time	1 (2.5%)	3 (5.5%)	4 (4.2%)
Missing	0 (0.0%)	1 (1.8%)	1 (1.1%)
Number of hours providing care in weekdays			
0–5	21 (52.5%)	8 (14.5%)	29 (30.5%)
6–10	8 (20.0%)	7 (12.7%)	15 (15.8%)
11–15	2 (5.0%)	12 (21.8%)	14 (14.7%)
Over 16	9 (22.5%)	22 (40%)	31 (32.6%)
Missing	0 (0.0%)	6 (10.9%)	6 (6.3%)
Number of hours providing care in weekend			
0–5	24 (60.0%)	4 (7.3%)	28 (29.5%)
6–10	7 (17.5%)	6 (10.9%)	13 (13.7%)
11–15	1 (2.5%)	10 (18.2%)	11 (11.6%)
Over 16	8 (20.0%)	29 (52.7%)	37 (38.9%)
Missing	0 (0.0%)	6 (10.9%)	6 (6.3%)
Age of the care recipients			
0–5	0 (0.0%)	25 (45.5%)	25 (26.3%)
6–10	0 (0.0%)	21 (38.2%)	21 (22.1%)
11–18	0 (0.0%)	6 (10.9%)	6 (6.3%)
60–69	6 (15.0%)	0 (0.0%)	6 (6.3%)
70–79	10 (25.0%)	0 (0.0%)	10 (10.5%)
80–89	15 (37.5%)	0 (0.0%)	15 (15.8%)
Over 90	7 (17.5%)	0 (0.0%)	7 (7.4%)
Missing	2 (5.0%)	3 (5.5%)	5 (5.3%)
Diagnoses of the care recipients for the caregiver group (multiple entries allowed)			
Dementia	22		
Parkinson's disease	2		
Stroke	2		
Diabetes	5		
Cardiovascular diseases	7		
Depression	4		
Other diagnoses	11		
Diagnoses of the care recipients for the parent group (multiple entries allowed)			
ADHD		27	
Autist spectrum disorder		9	
Learning disability		17	
Intellectual disability		2	
Mental disorders		2	
Other diagnoses		10	

**Figure 1:**
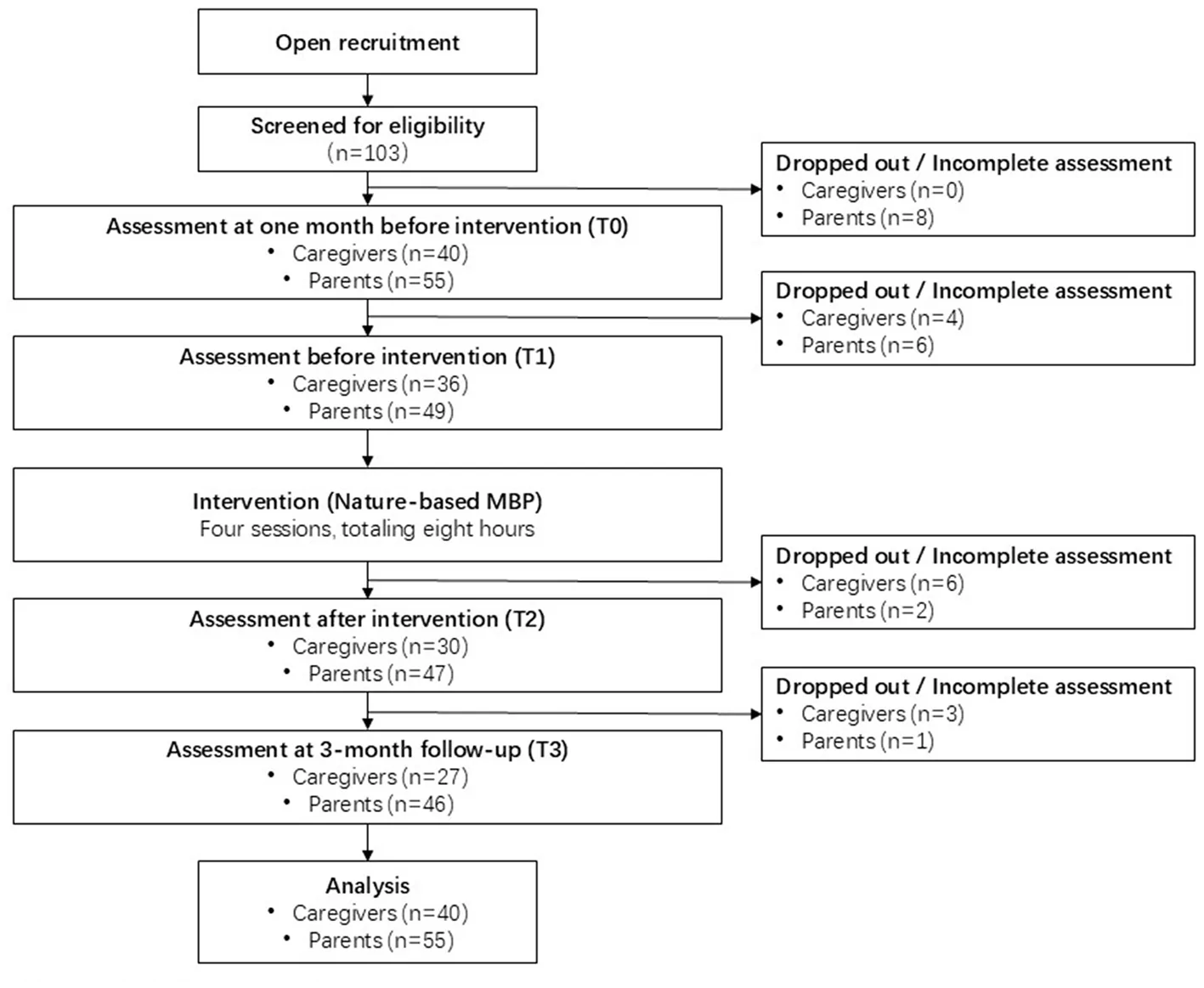
Participant flow chart.

#### Measures

**Center for Epidemiologic Studies Depression Scale** (CES-D; Radloff, 1977). The 10-item version of the CES-D was used to measure affective symptoms of depression. Example items include “I felt everything I did was an effort” and “My sleep was restless.” Participants rated the items on a 4-point Likert scale from 0 (*none of the time*) to 3 (*most of the time*). The CES-D demonstrated good internal consistency in this study, with a Cronbach's α of 0.836.

Subscales of anxiety and stress in the **Depression Anxiety Stress Scale** (DASS-A and DASS-S; Henry and Crawford, 2005). The DASS is a conventional set of instruments designed to measure depression, anxiety, and stress. In this study, the anxiety and stress subscales of the short version DASS-21 were used. Anxiety (7 items) refers to characteristics such as autonomic arousal and the subjective experience of anxious affect (e.g., “*I was worried about situations in which I might panic and make a fool of myself* ”), while stress (7 items) is sensitive to chronic non-specific arousal (e.g., “*I tend to over-react to situations*”). The Cronbach's α values were 0.897 for the Anxiety subscale and 0.899 for the Stress subscale, indicating good psychometric properties.

Physical distress and daily functioning in the **Body-Mind-Spirit Wellbeing Inventory** (BMSWBI-PD and BMSWBI-DF; Ng et al., 2005). The physical distress subscale has 14 items rated on an 11-point Likert scale to assess symptoms of physical distress, such as headache, chest pain, and fatigue. A score of 0 represents no distress, and a score of 10 represents extreme distress. The daily functioning subscale has 10 items that measure participants' evaluation of their daily functioning during the previous week, covering physical, cognitive, and motivational functions. Both subscales showed good internal consistency (Cronbach's α = 0.956 and 0.948, respectively).

**World Health Organization Wellbeing Index** (WHO-5; Heun et al., 1999). This measure includes five items that assess self-reported psychological wellbeing. Each item is scored from 5 (*all of the time*) to 0 (*none of the time*). The WHO-5 demonstrated good internal consistency, with a Cronbach's α of 0.931.

**The Family Adaptation, Partnership, Growth, Affection, Resolve Scale Family Functioning** (APGAR; Smilkstein et al., 1982) is a widely used self-reported measure of family functioning. It consists of five items that assess a parent's satisfaction with family functions across five domains: adaptation, partnership, growth, affection, and resolve. Responses are recorded on a 3-point scale, where 0 indicates “hardly ever”, and 2 indicates “almost always.” In this study, the APGAR Scale demonstrated strong psychometric properties, with a Cronbach's α of 0.958.

**Modified Conflict Tactics Scale** (CTS2; Straus, 1979). The Modified CTS2 is used to assess family conflict through self-reported measures of behavioral assault or psychological aggression among family members. The scale includes subscales for verbal aggression and physical conflict, which are common indicators of psychological and physical abuse. Participants are asked to indicate the frequency of such behaviors on a scale from 0 (*never happened*) to 4 (*happened more than 10 times in the past 6 months*). Two versions of the CTS2 were developed to address conflicts in parent-child relationships and caregiver-care recipient relationships, with Cronbach's α being 0.935 and 0.782 in this study, respectively.

**Self-Compassion Scale-Short Form** (SCS-SF; Raes et al., 2011). The SCS-SF measures the extent to which individuals treat themselves with compassion during difficult times. Participants rate their self-soothing and self-defeating attitudes on a five-point scale, ranging from 1 (*almost never*) to 5 (*almost always*). In this study, the SCS-SF demonstrated good reliability, with a Cronbach's α of 0.852.

**Connectedness to Nature Scale** (CNS; Mayer and Frantz, 2004). The CNS consists of 14 items rated on a 5-point Likert scale, from 1 (*strongly disagree*) to 5 (*strongly agree*). The CNS is used to assess whether participants feel more connected to nature after the program and whether they derive additional benefits from this connection. The scale showed good internal consistency, with a Cronbach's α of 0.870.

**Participation satisfaction**. An 8-item self-constructed program satisfaction scale was distributed to all participants along with other questionnaires. Sample items include “*This program helps me to identify strategies of emotional regulation in daily life*” and “*This program helps me to take care of my physical and psychological needs through practicing mindfulness*”. Participants rated their satisfaction on a 5-point scale from 1 (*strongly dissatisfied*) to 5 (*strongly satisfied*).

#### Qualitative study

Qualitative interviews were conducted to compare the quantitative and qualitative data. The aim was to provide an in-depth explanation of how nature-based mindfulness programs improved mental health and wellbeing by using the subjective experiences of participants. The interpretation of data would identify possible mechanisms based on the participants' perspectives and verify alignment with the three themes of attention restoration theory (Elliott et al., 2001).

The research team invited all participants who attended two or more sessions of the program to join a focus group interview. Independent interviewers, including the first author and his research assistant, who were not involved in program implementation, conducted the interviews. In these groups, participants were invited to share their experiences with the nature-based MBP. All focus groups were conducted 3 months after the program ended. Using a semi-structured protocol originally designed for program evaluation (Creswell and Creswell, 2017), the research team adapted the interviewing guide and conducted 10 focus groups with 34 participants. The interview protocol has included in Supplementary Materials section.

#### Data analysis

Using a mixed-methods approach, the research team analyzed both quantitative and qualitative data. Descriptive analysis was used to understand the participants' profiles. Paired-sample *t*-tests were conducted to investigate significant changes during the comparison period (T0 to T1), intervention period (T1 to T2), and maintenance period (T2 to T3). Hierarchical regression analysis was further conducted to explore if connectedness to nature at T1 and related variables could explain program outcomes. Given the number of statistical tests conducted across the measurements, a sensitivity analysis was further performed using a false discovery rate (FDR) correction to account for multiple comparisons, in line with the exploratory nature of the study. The FDR-corrected results are reported in the Supplementary Materials and referenced in the main manuscript. SPSS 24.0 was used for statistical analyses.

Qualitative analysis followed a deductive approach. A research assistant transcribed the interviews based on audio recordings. The researcher searched for evidence of the program, examined the connection between participants' experiences of change and the themes of attention restoration theory and connectedness with nature, and verified if they aligned with each other.

The application of mixed methods allowed for the use of qualitative findings to explain the quantitative results and to seek integration, clarification, and convergence of quantitative and qualitative data (Creswell and Creswell, 2017). While quantitative data highlighted the effects of nature on participant outcomes, qualitative data may supplement the possible change mechanism based on participants' perceptions about their benefits.

## Results

### Results from quantitative study

#### Outcomes and effect sizes

After FDR correction, changes from T0 to T1 in outcome measures were not statistically significant in the groups of both caregivers of old adults and parents of children (*p* > 0.05).

During the intervention period (T1 to T2), the caregiver group reported significant improvements in anxiety (*t* = 3.27, *p* = 0.017), stress (*t* = 3.29, *p* = 0.017), and wellbeing (*t* = −3.92, *p* < 0.001). Improvements were also observed in physical distress (*t* = 2.81, *p* = 0.033) and daily functioning (*t* = −2.61, *p* = 0.043). The improvement in connectedness with nature approached significance (*t* = −2.36, *p* = 0.061) after FDR correction.

Significant improvements from pre-test to follow-up were found in anxiety (*t* = 3.42, *p* = 0.017), stress (*t* = 3.04, *p* = 0.019), daily functioning (*t* = −2.76, *p* = 0.033), wellbeing (*t* = −3.32, *p* = 0.017), and connectedness with nature (*t* = −3.12, *p* = 0.017). Depression (*t* = 2.48, *p* = 0.054), physical distress (*t* = 2.39, *p* = 0.061), and family functioning (*t* = −2.29, *p* = 0.064) showed a trend of improvement, but they did not reach significance after correction. The details can be seen in the Table 3.

**Table 3:** Outcomes over time for caregiver of older adults (*n* = 40).

Variables	T0	T1	T2	T3	T0 vs. T1			T1 vs. T2			T1 vs. T3		
	M (SD)	M (SD)	M (SD)	M (SD)	*t*	*p*	*d*	*t*	*p*	*d*	*t*	*p*	*d*
Depression	10.93 (5.80)	11.61 (5.35)	10.47 (4.60)	9.41 (3.25)	−0.75	0.601	/	1.34	0.327	/	2.48	0.054	0.497
Anxiety	7.45 (8.98)	9.98 (8.32)	6.46 (6.48)	6.34 (6.37)	−1.97	0.116	/	3.27	0.017	0.473	3.42	0.017	0.491
Stress	12.85 (11.74)	16.07 (10.58)	11.25 (7.65)	11.42 (7.10)	−1.82	0.148	/	3.29	0.017	0.522	3.04	0.019	0.516
Physical distress	28.22 (26.29)	26.05 (24.40)	16.38 (12.82)	18.10 (14.36)	0.63	0.649	/	2.81	0.033	0.496	2.39	0.061	0.397
Daily functioning	59.20 (20.51)	57.39 (18.34)	63.18 (16.52)	64.77 (17.00)	0.59	0.662	/	−2.61	0.043	0.332	−2.76	0.033	0.417
Wellbeing	13.03 (4.74)	13.33 (5.31)	15.87 (5.18)	16.24 (5.59)	−0.38	0.754	/	−3.92	< 0.001	0.484	−3.32	0.017	0.534
Family functioning	5.38 (3.22)	5.78 (2.72)	6.00 (2.42)	6.80 (2.46)	−0.97	0.484	/	−0.65	0.649	/	−2.29	0.064	0.393
Psychological abuse	3.53 (2.85)	3.22 (2.85)	3.37 (2.30)	2.60 (2.34)	1.04	0.455	/	−0.31	0.782	/	1.43	0.297	/
Physical abuse	0.75 (1.41)	0.81 (1.68)	1.80 (2.72)	0.96 (1.87)	0.19	0.853	/	−2.13	0.086	0.438	−0.48	0.695	/
Self-compassion	34.13 (9.59)	32.83 (8.08)	31.00 (10.09)	31.35 (7.01)	0.91	0.507	/	1.28	0.342	/	1.09	0.442	/
Connection with nature	44.35 (11.01)	43.55 (8.37)	46.00 (6.07)	48.42 (8.48)	0.50	0.695	/	−2.36	0.061	0.335	−3.12	0.017	0.578

*p*-values were after FDR correction.

The effect size of improvement for caregivers of older adults in depression was small (*d* = 0.497), while other effect sizes ranged from small to moderate (anxiety, stress, physical distress, daily functioning, wellbeing, connectedness with nature, family functioning), ranging from *d* = 0.332 to 0.578.

During the intervention period (T1 to T2), the parent group showed significant improvements were observed in wellbeing (*t* = −3.19, *p* = 0.010). Stress (*t* = 2.39, *p* = 0.072), physical distress (*t* = 2.25, *p* = 0.080), daily functioning (*t* = −2.39, *p* = 0.072), and self-compassion (*t* = 2.32, *p* = 0.072) approached significance.

From pre-test to follow-up (T1 to T3), significant improvements were found in anxiety (*t* = 3.50, *p* = 0.007), stress (*t* = 4.45, *p* < 0.001), physical distress (*t* = 3.31, *p* = 0.010), psychological aggression (*t* = 3.88, *p* < 0.001), corporate punishment (*t* = 4.31, *p* < 0.001), and physical maltreatment (*t* = 3.95, *p* < 0.001). Depression (*t* = 2.60, *p* = 0.054) and daily functioning (*t* = −2.34, *p* = 0.072) approached significance, as shown in Table 4.

**Table 4:** Outcomes over time for parents (*n* = 55).

Variables	T0	T1	T2	T3	T0 vs. T1			T1 vs. T2			T1 vs. T3		
	M (SD)	M(SD)	M(SD)	M(SD)	*t*	*p*	*d*	*t*	*p*	*d*	*t*	*p*	*d*
Depression	17.02 (6.12)	16.10 (5.76)	16.00 (5.24)	13.96 (5.29)	1.51	0.260	/	0.16	0.928	/	2.60	0.054	0.387
Anxiety	16.51 (10.36)	15.79 (9.39)	14.68 (7.68)	11.16 (8.41)	0.60	0.707	/	1.02	0.451	/	3.50	0.007	0.519
Stress	23.45 (10.79)	24.01 (10.71)	20.91 (8.41)	17.83 (10.11)	−0.54	0.734	/	2.39	0.072	0.322	4.45	0.000	0.593
Physical distress	47.91 (25.34)	54.71 (30.16)	46.41 (23.11)	42.45 (26.42)	−2.19	0.082	0.244	2.25	0.080	0.309	3.31	0.010	0.432
Daily functioning	49.82 (16.88)	47.11 (16.54)	53.17 (16.55)	52.24 (15.34)	1.51	0.260	/	−2.39	0.072	0.366	−2.34	0.072	0.322
Wellbeing	12.55 (6.59)	11.82 (6.63)	14.83 (6.65)	11.85 (4.74)	1.27	0.350	/	−3.19	0.010	0.453	−0.03	0.974	/
Family functioning	6.13 (4.62)	5.90 (4.66)	6.43 (3.78)	4.87 (2.31)	0.81	0.584	/	−1.11	0.426	/	1.64	0.229	/
Psychological aggression	7.33 (4.46)	6.77 (4.07)	6.19 (3.76)	4.91 (3.35)	1.67	0.227	/	1.46	0.268	/	3.88	< 0.001	0.499
Corporate punishment	5.54 (3.81)	4.90 (3.61)	4.81 (2.97)	2.89 (2.48)	2.10	0.098	0.172	0.25	0.928	/	4.31	< 0.001	0.649
Physical maltreatment	2.74 (2.89)	2.52 (2.69)	2.63 (2.13)	1.33 (1.71)	1.04	0.451	/	−0.39	0.841	/	3.95	< 0.001	0.528
Self-compassion	35.85 (7.47)	36.00 (7.07)	33.54 (6.11)	35.83 (6.32)	−0.16	0.928	/	2.32	0.072	0.372	0.20	0.928	/
Connectedness to nature	43.36 (9.59)	43.51 (10.10)	44.88 (9.46)	44.28 (7.71)	−0.12	0.934	/	−1.26	0.350	/	−0.66	0.681	/

*p*-values were after FDR correction.

The effect size of improvement for the parent group in corporate punishment was moderate (*d* = 0.649), while those for other outcomes (physical distress, depression, anxiety, stress, psychological aggression, daily functioning, and physical maltreatment) showed small to moderate effect sizes (*d* ranging from 0.309 to 0.593). The changes of selected outcomes over time is illustrated in Figures 2–7.

**Figure 2:**
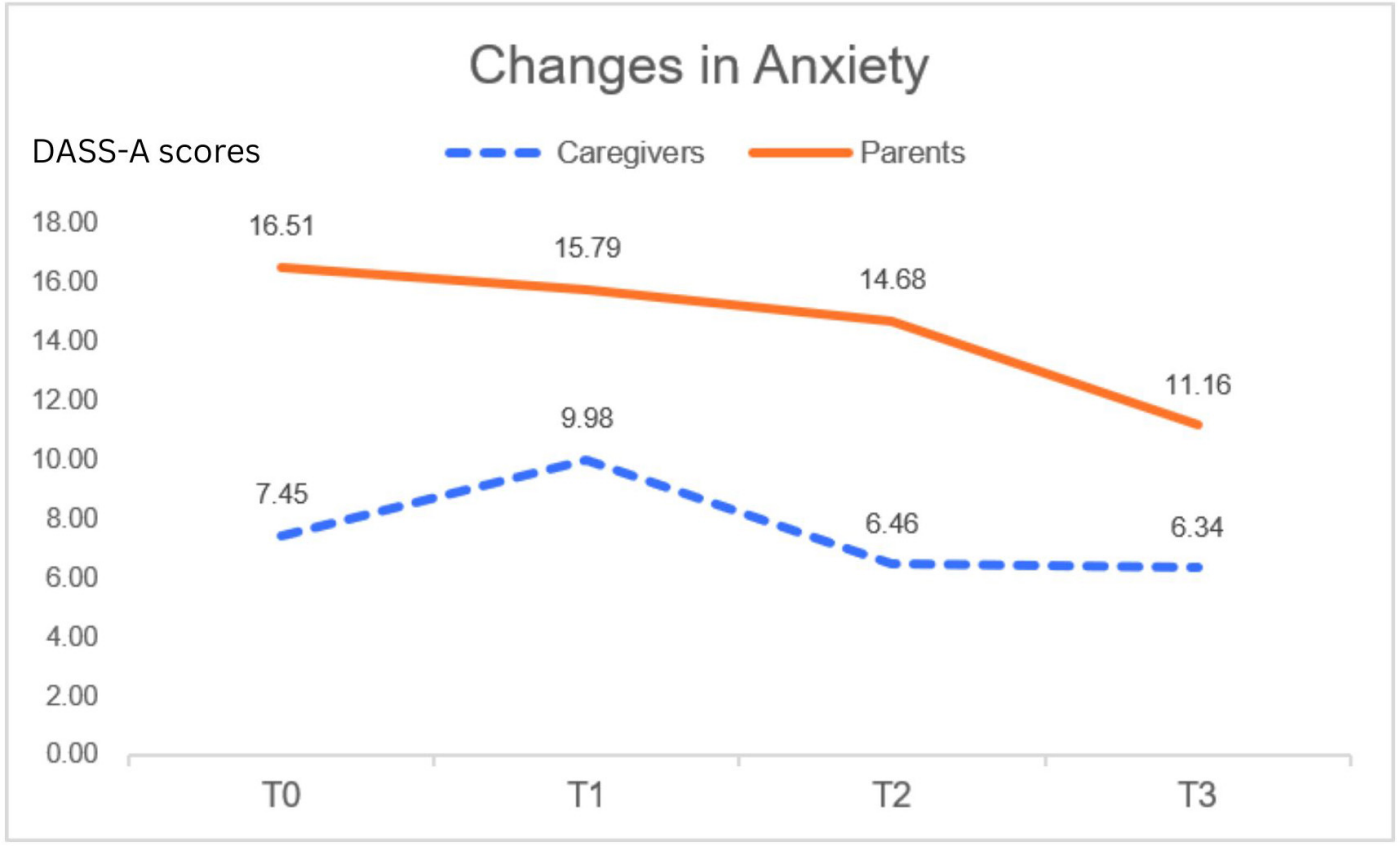
Changes in anxiety. Caregivers showed significant changes from T1 to T2 and from T1 to T3. Parents showed significant changes from T1 to T3.

**Figure 3:**
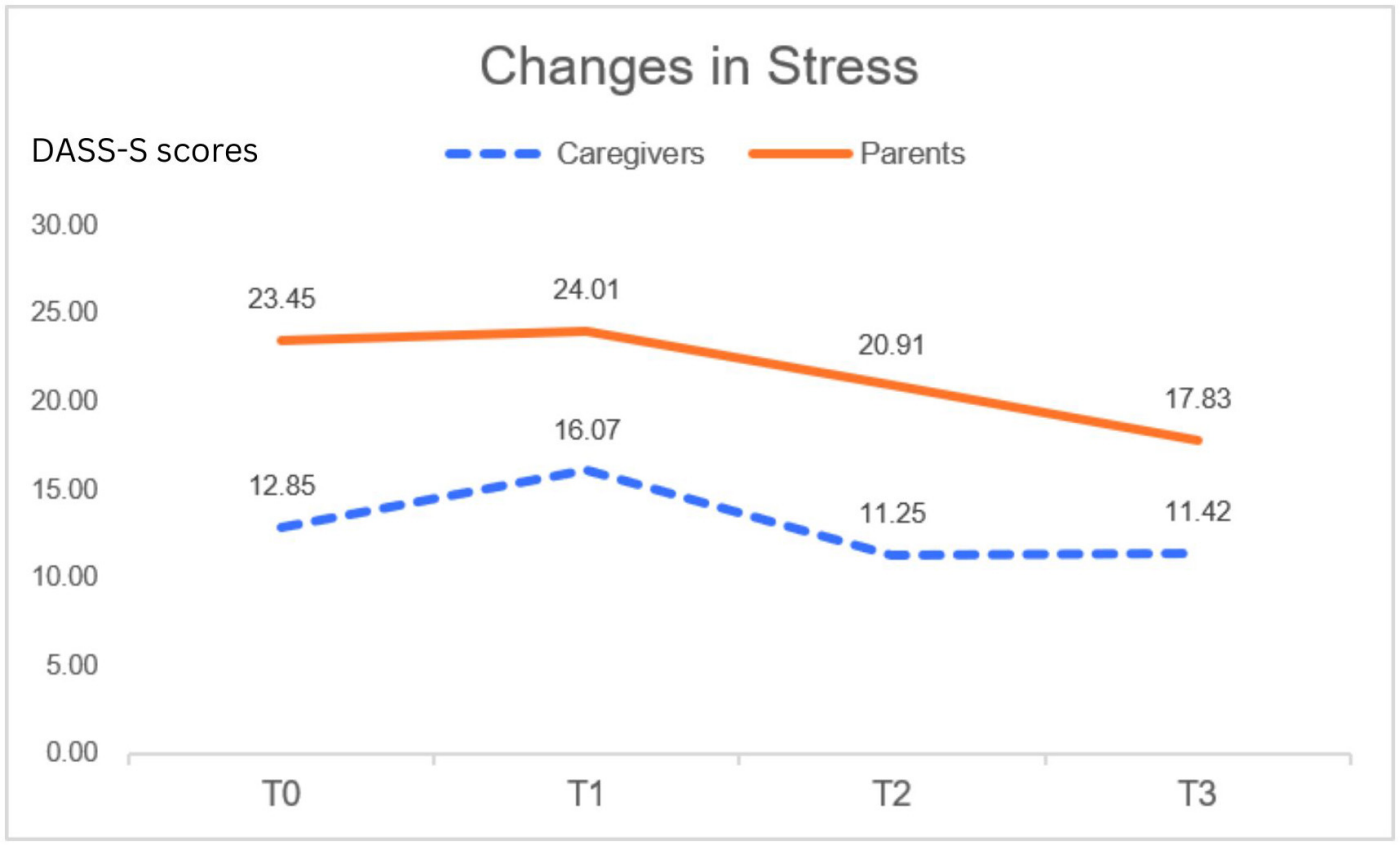
Changes in stress. Caregivers showed significant changes from T1 to T2 and from T1 to T3. Parents showed significant changes from T1 to T3.

**Figure 4:**
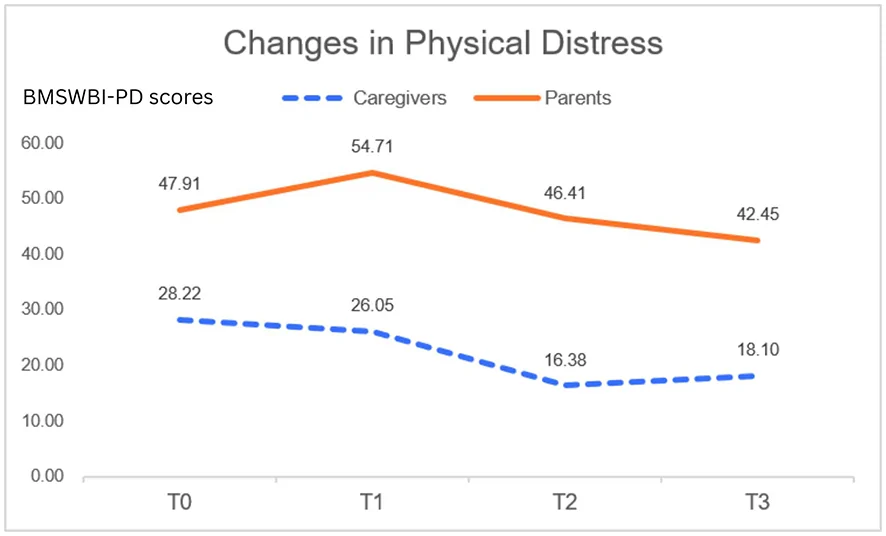
Changes in physical distress. Caregivers showed significant changes from T1 to T2. Parents showed significant changes from T1 to T3.

**Figure 5:**
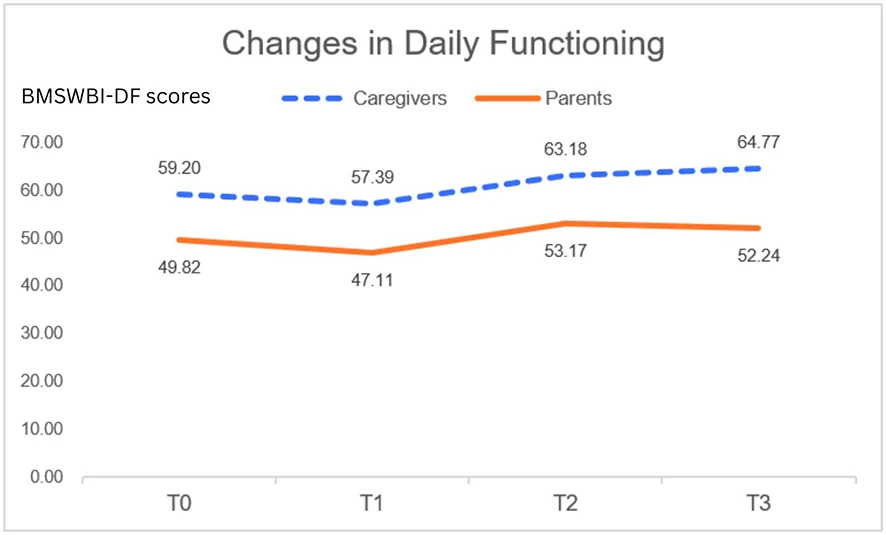
Changes in daily functioning. Caregivers showed significant changes from T1 to T2 and from T1 to T3. No significant changes were observed in parents.

**Figure 6:**
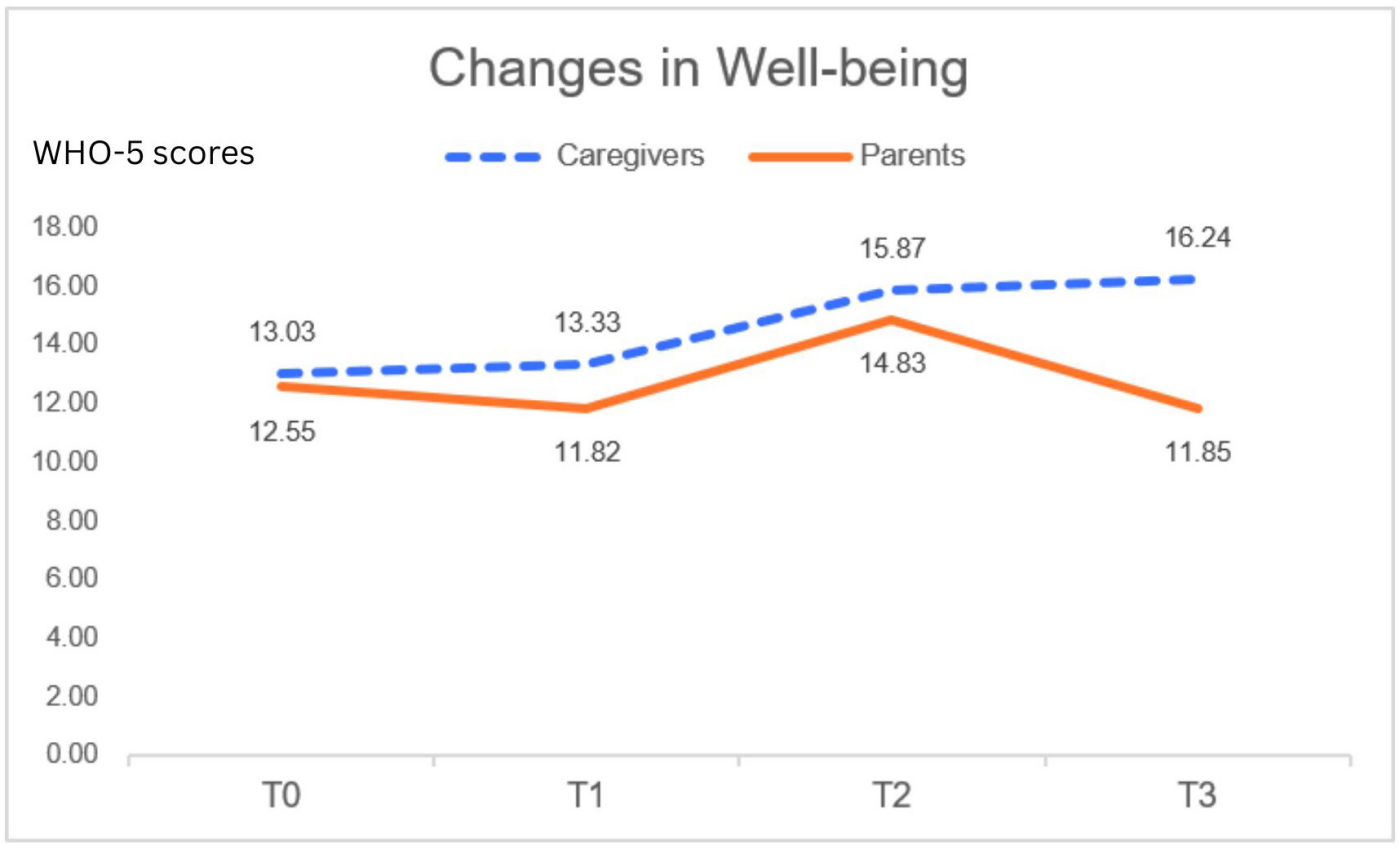
Changes in wellbeing. Caregivers showed significant changes from T1 to T2 and from T1 to T3. Parents showed significant changes from T1 to T2.

**Figure 7:**
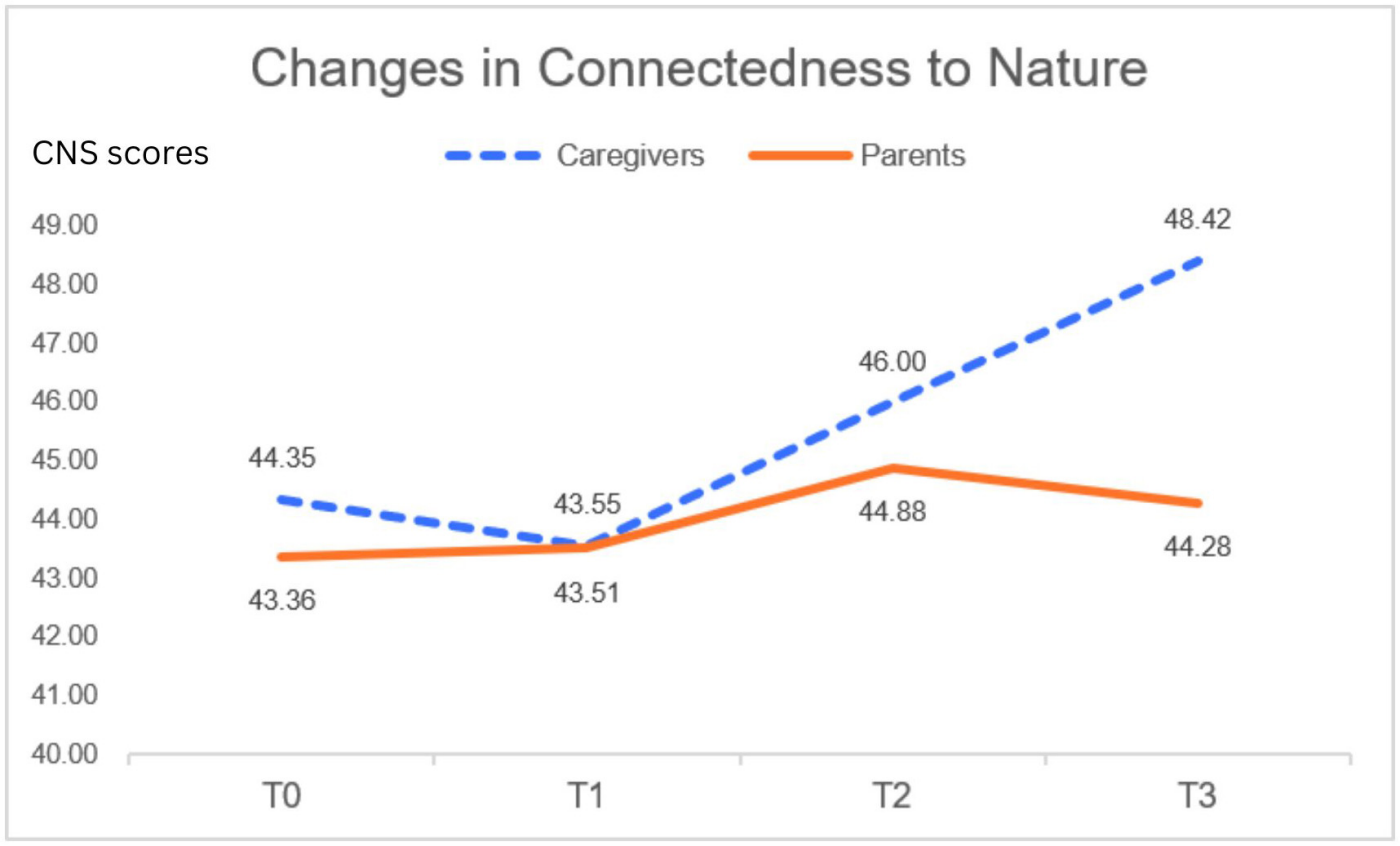
Changes in connectedness to nature. Caregivers showed significant changes from T1 to T3. No significant changes were observed in parents.

#### The role of nature in the outcome

The time spent at the family retreat center during the program was 4.51 ± 3.10 h for caregivers and 4.28 ± 2.18 h for parents. Overall, the average time spent in nature was 4.38 ± 2.59 h.

Hierarchical regression analysis was conducted to investigate the role of nature in program outcomes. After controlling for the basic information of caregivers (age, gender, education, marriage status, religion, working status, living status) and their primary outcome of depression at T1, which were considered as potential co-variants, connectedness to nature at T1 and hours spent at the retreat center explained 46.1% of the variance in changes in depression, 20.3% of the variance in family functioning, and 30.6% of the variance in family conflicts at post-intervention. Connectedness to nature significantly associated changes in depression at T2 (β = −0.285, *t* = −3.205, *p* = 0.002).

After controlling basic information of caregivers and their primary outcome of depression at T1, connectedness to nature at T1 and hours spent at the retreat center explained 47.8% of the variance in changes in depression, 30.5% of the variance in family functioning, and 36.6% of the variance in family conflicts at follow-up. Connectedness to nature significantly associated with changes in depression (β = −0.236, *t* = −2.721, *p* = 0.008) and family functioning (β = 0.442, *t* = 4.421, *p* < 0.001) at T3. The effect of hours spent at the retreat center was analyzed, but no significant results were found.

#### Attendance and participation satisfaction

Based on the records of 95 participants, 11 parents and caregivers attended only one session. For the remaining participants who attended at least two sessions and were considered completers of the nature-based MBP, the mean attendance in this 4-session program was 3.37 ± 0.78 sessions.

Scores from the satisfaction survey ranged from 4.15 ± 0.74 to 4.54 ± 0.59 on a 5-point scale. Specifically, item 2, “*This program helps me to take care of my physical and psychological needs through practicing mindfulness*,” received a score of 4.35 ± 0.67. Item 8, “*Overall, I feel satisfied with the program*,” received a score of 4.54 ± 0.59. Both attendance and satisfaction survey results indicated a high level of participant satisfaction.

#### Qualitative study

A total of 10 focus groups were organized, with 34 program completers participating 3 months after the nature-based MBP. Among them, 14 were caregivers of older adults, while 20 were parents. Transcriptions of participants' subjective experiences were collected to illustrate the perceived benefits of the nature-based mindfulness program. After reviewing the quantitative study results, the research team decided to focus on participants' perceptions of the program's benefits and the role of nature in the overall positive outcomes. The transcriptions were initially reviewed by the first author, with coding inspired by Kaplan's attention restoration theory (Kaplan, 1995). The coding results were shared with two co-authors and finalized after incorporating their feedback.

***Being away and soft fascination***: Visits to the family retreat center provided physical distance from daily environments. Many parents and caregivers appreciated that the program and natural setting offered a space to relieve caregiver stress and burden. Participants reported restored focus and mental capacity, with reduced fatigue as their attention was engaged by nature and its aesthetics. The following quotations illustrate the theme of being away and soft fascination:

“Take your time eating, there's no rush at all. The pace is slow, very slow, and comfortable. Everything slows down, slowly savoring the fruits. I brought along those delicious tuna sandwiches myself. I‘ve never had tuna that tasted so good before... Even when I've had it in the past, it didn't seem as delicious. That day, it felt exceptionally tasty... It was all so comfortable, so sweet. That's how I remember enjoying that journey...” (Group 2, Caregiver, L)

“I used to have a lot of pain in my hand, and I couldn't carry the food when I visited the market properly. But after I learned to relax, I realized that maybe I had been tensing my muscles too tightly. Learning to relax actually made the pain less intense. At least now I don't experience pain all the time... and even my headaches have changed. In the past, my headaches would make my head feel like it was about to explode, but now they're less severe... I used to rely on headache medication, taking two pills every night and day just to relieve the pain and get some sleep. But now, I need them less frequently. I don't have to take headache medication anymore.” (Group 4, Parent, Y)

“Arriving at that environment indeed helps people relax. When you enter a peaceful place, the White House (name of the retreat center), it changes the mood... Visual experiences are different, you know. It feels very comforting... Especially when I take a leisurely walk in the garden, taking small steps and enjoying the surroundings.” (Group 1, Caregiver, H)

“When I enter that environment, I feel so comfortable, as if I've immersed myself in nature. Being surrounded by flowers and plants, when we go for a walk in the flower garden and get to know different types of flora, seeing various flowers displayed together, it brings a sense of tranquility. Sitting on a bench, I feel like closing my eyes and practicing mindfulness, taking in the scent of nature, feeling the air and the gentle breeze brushing against me.” (Group 4, Parent, Y)

“Being in the White House feels like completely detaching from everything in the present moment. It provides a space for me to catch my breath and feel more comfortable. There are multiple spots where I can take a deep breath. Sometimes, I even realize that I forget to breathe in certain situations. Participating in this activity helps me find the root causes of certain issues. In the white room, I have found a breathing space. The reason for bringing back a flower is to evoke the feeling of being in that space. It's a feeling where I can let go of everything, not needing to think about anything, and completely focus on myself.” (Group 7, M)

***Extent***: Parents and caregivers experienced a sense of relatedness, associating their experiences in natural environments with their parenting and caregiving roles. They felt relaxed and enjoyed the program offered at the family retreat center, nurtured by the natural environment and mindfulness practices. The following quotes were identified under the theme of extent:

“I feel like it was helpful during that period of time, my mood relaxed a lot. The environment, the air, and engaging in those activities, listening to mindfulness music, made me feel much more comfortable. But after some time, it seemed to fade away, and I feel like I've become lazy again.” (Group 1, Caregiver, L)

“It seems like you felt a strong connection with nature and that it had a positive impact on your mindset. Being in tune with nature can indeed help to relax and uplift one's spirits. When you are burdened with worries or feeling down, immersing yourself in that environment can truly have a positive effect on your wellbeing. Looking at the surroundings and being in touch with nature can be incredibly uplifting.” (Group 9, Caregiver, 8)

“Before participating, there were times when I would forget to breathe and even experience a sense of emotional numbness. It's like feeling disconnected and suddenly bursting into tears without any apparent reason. Perhaps there were suppressed emotions that I wasn't aware of. During the process of participation, I was able to gain some insights into the issues…” (Group 7, Parent, M)

“Indeed, personally connecting with nature and practicing mindfulness in that setting can be highly effective. Nature has its unique sounds, such as birds singing, and one can experience the presence of trees while breathing in the refreshing air. When one participates in mindfulness activities in the White House, it can complement the experience by aligning with the theme.” (Group 10, Parent, 4)

***Compatibility***: This theme suggests that natural environments are well suited to individuals' preferences or purposes in parenting and family caregiving. Integrating natural environments with mindfulness practices can support parents and caregivers and enhance their adaptive coping. The following quotes illustrate the theme of compatibility:

“I picked up the falling leaves, some were yellow, some were green, but they all ended up on the ground. It's like life itself. Every action we take is akin to cultivating and nurturing, starting from a state of unfamiliarity and gradually adapting and becoming familiar. It's as if changes in life instill a sense of belief in us.” (Group 4, Parent, Y)

“It's important to relax and take care of yourself first. If you feel too tense, it can be challenging to manage everything. Additionally, it's essential not to let your own emotions dictate how you care for your children. Avoid putting too much pressure on yourself and learn not to accommodate all of their demands. In the past, you may have felt the need to constantly accommodate them, but now it's crucial to prioritize self-care and not solely rely on being their mother to fulfill their every need.” (Group 4, W)

“If there is an activity I can participate in, it would feel like finding a hiding place where I can take a break. It's something I wouldn't normally pay attention to, but it's a way to divert my attention and prevent myself from obsessing over things. It's important not to get fixated and to avoid getting worked up over minor issues. I believe this kind of activity can bring significant benefits to me.” (Group 5, Parent, M)

“When we went to the White House, because we didn't bring the children along, I felt truly amazing. The previous activities, I did them with my child, and it felt different. It was so relaxing. When I walked out to the garden and breathed in the free air, it felt so liberating. That's when I realized that I must give myself more opportunities to de-stress. When we went that day, we were incredibly happy, breathing in the free air. The difference I felt was that I could slow down my pace and appreciate myself more, and be grateful to myself. There are many things that I need to learn to let go. My child has grown up, today was their first day of middle school, and compared to before, I feel the need to take care of myself more. Don't spend too much time focusing solely on the children.” (Group 7, Parent, 3)

“Sometimes, when I feel tense, it first manifests as a pain in my chest, and then my whole body becomes stiff. Over time, I have come to understand my body better, and I have started to have a better understanding of my condition. I have learned to accept it. Even if doctors couldn't provide me with an answer, if that's the case, then I accept it. You have to work with it and coexist with it. It has given me a new perspective in terms of my mental and emotional wellbeing.” (Group 7, Parent, M)

“I have learned that through mindful walking, we can truly appreciate nature. Many things go unnoticed in our daily lives, but when we walk slowly and mindfully, our hearts become calm. When we're in a hurry, we tend to walk fast, even taking less than five steps before picking up the pace again. However, when you walk slowly and mindfully, you realize that it is actually very comfortable. It teaches us not to rush and to take our time.” (Group 9, Caregiver, 3)

## Discussion

Hypothesis 1 is largely supported. Parents and caregivers indicated stable mental health and psychosocial status before the program commenced. In contrast, significant changes in many outcome measures in this study appeared after T1, and the changes that emerged after T1 were likely to be the effect of the program. Additionally, hypotheses 2 and 3 are mostly supported. Participants in the caregiver group reported significant improvements in anxiety and stress, with the effects maintained at follow-up. As for depression, the caregivers of old adults did not show a significant decrease at post-intervention, but the change in depression from pre-intervention to follow-up approached significance. Based on the outcome measures over time, it is likely that the improved mental health outcomes for caregivers are associated with the nature-based MBP. These findings align with recent reviews of MBPs for caregivers of dementia (Han, 2022) and those with chronic diseases (Erdogan Yüce et al., 2024). However, it should be emphasized that many studies in these reviews were based on relatively intensive MBPs similar to MBSR and MBCT. Another study focusing on a low-intensity MBP for caregivers of frail older adults found improvements in depression during the follow-up period but not post-intervention, with no improvement in anxiety or caregiving burden (Zhang et al., 2025). The favorable and enhanced outcomes in caregiver mental health in this study may be attributed to the effects of nature (Chang et al., 2024). Unfortunately, the study design did not include a control group, and the caregiver profile was relatively heterogeneous. Future studies should further investigate the effects of nature-based MBPs on caregivers using randomized controlled trials and more restricted participant criteria.

Conversely, parents reported significant improvement in wellbeing at post-intervention, while stress approached significance. From pre-intervention to follow-up, parents reported significant improvements in anxiety and stress, while depression approached significance. Most MBPs for parents have used parenting stress and other child-related variables as outcomes, which were not selected in our study. Variations in study and program design for parent MBPs and outcome measures create challenges in comparing results across studies (Caetano et al., 2024). It should be emphasized that only a few studies of MBPs for parents have reported improvements in depression (Lo et al., 2017; Potharst et al., 2019), while other studies did not find similar positive changes (Cheung et al., 2022; Lyu and Lu, 2023), suggesting that parents' depressive symptoms may be related to more complex factors. Future studies should include more parent outcomes and further explore how nature-based MBPs can promote parent mental health using rigorous study designs.

In this study, we further explored the effects of nature-based MBPs in promoting psychosocial functioning for parents and caregivers. Previous studies of nature-based MBPs have focused on outcomes related to mental health and wellbeing (Djernis et al., 2019; Vecchio et al., 2026). This study has further provided additional evidence, showing significant improvements in physical distress and marginally significant improvements in daily functioning, and expanding the target population from students to other disadvantaged groups. Recently, more studies have explored the role of nature in supporting individuals with various mental health conditions (Hoenders et al., 2018; Hood and Baumann, 2024; Watkins-Martin et al., 2022). Future studies should explore whether nature-based MBPs can benefit special populations, such as children and adults diagnosed with major depressive disorders, attention deficit/hyperactivity disorder, or psychosis.

One more interesting finding in this study is the parallel changes of connectedness to nature with family conflicts, particularly improvements in psychological aggression, corporate punishment, and physical maltreatment in the parent group. Few studies have applied MBPs to prevent domestic violence, mostly incorporating other intervention strategies such as acceptance and commitment therapy (Mitchell and Wupperman, 2023). Nesset et al. (2019) investigated the use of MBSR as a treatment for domestic violence from a forensic referral source and found similar positive outcomes with cognitive behavioral therapy. It should be emphasized that in this study, the frequency of conflicts and aggression at baseline was relatively low. Future studies should explore the effects of low-intensity MBPs on parents and caregivers at risk of domestic violence, using a randomized controlled group design and compare the effects of MBPs with and without nature-based practice.

Hypothesis 4 in this study is supported. Regression analysis results showed that enhanced connectedness to nature and time spent in nature associated with improvements in depression, family functioning, and family conflicts. Connectedness to nature after-program associated with multiple outcomes in our study, although such relationship was correlational only, due to the limitations of a quasi-experimental study design. In qualitative analysis, participants' perceived benefits of the program including helping them to relieve fatigue and caregiving burden, gaining awareness and insights gained from mindfulness practice and family care, and improving self-care and adaptive coping (Kaplan, 1995).

The study strongly advocates for the integration of nature into MBPs to enhance mental health and wellbeing. By combining mindfulness practices with natural environment and exploring various program structures, the diverse needs of target populations can be better addressed. The intervention protocol in this study included nature-based mindfulness practice as a minor component. Considering the promising benefits of nature, alternative formats of nature-based mindfulness programs, such as a mindful day in nature, or a standard 8-week mindfulness program with nature-based sessions could be developed. Moreover, to accommodate parents and caregivers who face challenges due to care responsibilities, parallel programs tailored for children and older adults can be introduced. These parallel programs aim to support and encourage the commitment of parents and caregivers to engage in a nature-based mindfulness program.

More nature-based facilities should be integrated into community planning and facilities to support parents and caregivers, providing them with opportunities to prioritize self-care in their daily lives. Recent studies indicate that virtual reality combined with an 8-week MBP was feasible (Choe et al., 2020). Such creative format can be particularly beneficial for parents and caregivers who are unable to access natural settings. Other participants may prefer a more immersive experience in nature could benefit from activities like forest walking, and integrating mindfulness practice with horticultural therapy. These practices can enhance mindfulness and self-awareness, offering parents and caregivers a reprieve from their daily caregiving responsibilities (Burger et al., 2024). Overall, adopting a personalized and diverse approach in developing alternative MBPs may boost motivation and engagement in practicing mindfulness.

Recent theoretical and empirical work has highlighted the importance of meaning in life as a potential mediator of the benefits of mindfulness-based interventions. For example, Ehmann and Gawrysiak (2025) suggested that mindfulness may enhance psychological wellbeing in part by increasing a sense of meaning in life. Furthermore, the Mindfulness-to-Meaning Theory posits that mindfulness can foster meaning through cognitive processes such as decentering, savoring, and attentional broadening, which may be further supported by experiences in nature (Garland and Fredrickson, 2019). Future research on nature-based mindfulness programs might consider examining meaning in life as an outcome and a potential factor in the mechanism, which may provide deeper insight into how these interventions promote wellbeing.

Despite the positive results, the authors acknowledge the following limitations of the study and provide directions for further research. First, the study did not include a control group. Although the time series design allows us to infer improvements in participants due to the intervention using a single group design, future studies should consider including a control group to verify the program effect. Additionally, a two- or multi-arm design with a sufficiently large sample size can allow investigators to conduct mediation analysis and detect the effect of nature in the intervention. Second, the study relies solely on self-reported measures. Nature-based interventions make blinding participants impossible. Future studies may consider using objective measures such as biomarkers like salivary cortisol or heart rate variability to strengthen the evidence for nature-based MBPs. Third, the study did not include a direct measure of mindfulness, which can be one of the central constructs in the intervention. This decision was made to reduce participant burden and avoid survey fatigue, given the number of other validated measures included. However, the absence of a mindfulness measure might limit our ability to directly assess changes in mindfulness. Future research should consider incorporating validated mindfulness scales to directly measure the mindfulness status. Fourth, the study did not include booster sessions or further MBPs after the initial sessions. In focus group interviews, one participant expressed concern about maintaining regular mindfulness practice after the program, indicating a need for ongoing support to sustain the program's effects for parents and caregivers. Lastly, the sample was dominated by female, and less than 10% of the participants were male. In addition, married participants were over 70%, and there were substantial underrepresentation among participants with other marital status (single, divorced/separated, and widowed). Parents and caregivers with these characteristics may not respond to the program in a similar way. Future studies should explore the impact of nature-based mindfulness programs among these special populations. Furthermore, a randomized controlled trial with rigorous design is recommended to strengthen the preliminary evidence in this study, and verify the benefits of nature-based mindfulness programs on parents and caregivers.

## Data Availability

The raw data supporting the conclusions of this article will be made available by the authors, without undue reservation.
