# Supplementary material for: Nature-based mindfulness program for parents and caregivers: a mixed-methods study
Source: Front Psychol. 2026 Oct 15;17:1900785. doi: 10.3389/fpsyg.2026.1900785 (PMC13716632; doi:10.3389/fpsyg.2026.1900785)
Supplement: Supplementary file 1 [file Supplementary_file_1.docx]

Supplementary information

Appendix 1 Focus group interview guide

Opening address:

Thank you all for taking the time to participate in today's focus group. As a member of this research project team, the main purpose of our focus group is to understand your journey in participating in the "Nature-Based Mindfulness for Well-being" group and the impact it has had on you. Each participant will have the opportunity to share their experiences.

About seeking help

1.1. What motivated you to join this project? How would you describe yourself as a parent/caregiver before participating in this project?

1.2. What kind of services have you been seeking since experiencing emotional stress or burden? (Including self-help, individual counseling, group counseling, medication)

1.3. Have these services or programs (including individual and group counseling) been helpful to you?

About personal changes

2.1. What have you learned or experienced since you participated in the parent/caregiver activities at the Family Retreat Centre? Have you noticed any changes in yourself? (For example: Have your behaviors, feelings, or thoughts changed compared to before? What specific things have you gained from this program?)

2.2. Is there something you wanted to change but have not been able to since joining this group? If yes, please share up to three of the most significant changes you have experienced since joining this group.

Regarding this activity, what has been obstructive, unhelpful, negative, or disappointing to you? Please provide examples, specific aspects, or events. Is there any missing content in the program? What could make the program more effective or helpful?

About recommendations

5.1. Do you have any suggestions for us regarding the caregiver activities? Is there anything else you would like to tell us?
