# Supplementary material for: Nature-based mindfulness program for parents and caregivers: a mixed-methods study
Source: Front Psychol. 2026 Oct 15;17:1900785. doi: 10.3389/fpsyg.2026.1900785 (PMC13716632; doi:10.3389/fpsyg.2026.1900785)
Supplement: Supplementary file 2 [file Table_1.docx]

**Supplementary Material**

Appendix 1. Outcomes over time for caregiver of older adults before and after Benjamini-Hochberg False Discovery Rate (FDR) correction

| Variables | T0 vs T1 | | | T1 vs T2 | | | T1 vs T3 | | |
| --- | --- | --- | --- | --- | --- | --- | --- | --- | --- |
|  | *t* | *p* | *q* | *t* | *p* | *q* | *t* | *p* | *q* |
| Depression | -0.75 | 0.455 | 0.601 | 1.34 | 0.188 | 0.327 | 2.48 | 0.018 | 0.054 |
| Anxiety | -1.97 | 0.056 | 0.116 | 3.27 | 0.002 | 0.017 | 3.42 | 0.001 | 0.017 |
| Stress | -1.82 | 0.076 | 0.148 | 3.29 | 0.002 | 0.017 | 3.04 | 0.004 | 0.019 |
| Physical distress | 0.63 | 0.531 | 0.649 | 2.81 | 0.008 | 0.033 | 2.39 | 0.022 | 0.061 |
| Daily functioning | 0.59 | 0.562 | 0.662 | -2.61 | 0.013 | 0.043 | -2.76 | 0.009 | 0.033 |
| Well-being | -0.38 | 0.708 | 0.754 | -3.92 | 0.000 | 0.000 | -3.32 | 0.003 | 0.017 |
| Family functioning | -0.97 | 0.337 | 0.484 | -0.65 | 0.522 | 0.649 | -2.29 | 0.027 | 0.064 |
| Psychological abuse | 1.04 | 0.303 | 0.455 | -0.31 | 0.758 | 0.782 | 1.43 | 0.162 | 0.297 |
| Physical abuse | 0.19 | 0.853 | 0.853 | -2.13 | 0.039 | 0.086 | -0.48 | 0.632 | 0.695 |
| Self-compassion | 0.91 | 0.369 | 0.507 | 1.28 | 0.207 | 0.342 | 1.09 | 0.281 | 0.442 |
| Connection with nature | 0.50 | 0.618 | 0.695 | -2.36 | 0.024 | 0.061 | -3.12 | 0.003 | 0.017 |

Note: *q*-values represent FDR-adjusted *p*-values. Significance after correction is denoted as *q* < 0.05.

Appendix 2. Outcomes over time for parents before and after Benjamini-Hochberg FDR correction

| Variables | T0 vs T1 | | | T1 vs T2 | | | T1 vs T3 | | |
| --- | --- | --- | --- | --- | --- | --- | --- | --- | --- |
|  | *t* | *p* | *q* | *t* | *p* | *q* | *t* | *p* | *q* |
| Depression | 1.51 | 0.137 | 0.260 | 0.16 | 0.870 | 0.928 | 2.60 | 0.012 | 0.054 |
| Anxiety | 0.60 | 0.550 | 0.707 | 1.02 | 0.313 | 0.451 | 3.50 | 0.001 | 0.007 |
| Stress | -0.54 | 0.591 | 0.734 | 2.39 | 0.020 | 0.072 | 4.45 | <0.001 | 0.000 |
| Physical distress | -2.19 | 0.032 | 0.082 | 2.25 | 0.029 | 0.080 | 3.31 | 0.002 | 0.010 |
| Daily functioning | 1.51 | 0.137 | 0.260 | -2.39 | 0.020 | 0.072 | -2.34 | 0.023 | 0.072 |
| Wellbeing | 1.27 | 0.210 | 0.350 | -3.19 | 0.002 | 0.010 | -0.03 | 0.974 | 0.974 |
| Family functioning | 0.81 | 0.422 | 0.584 | -1.11 | 0.272 | 0.426 | 1.64 | 0.108 | 0.229 |
| Psychological aggression | 1.67 | 0.101 | 0.227 | 1.46 | 0.149 | 0.268 | 3.88 | <0.001 | 0.000 |
| Corporate punishment | 2.10 | 0.041 | 0.098 | 0.25 | 0.801 | 0.928 | 4.31 | <0.001 | 0.000 |
| Physical maltreatment | 1.04 | 0.304 | 0.451 | -0.39 | 0.701 | 0.841 | 3.95 | <0.001 | 0.000 |
| Self- compassion | -0.16 | 0.876 | 0.928 | 2.32 | 0.024 | 0.072 | 0.20 | 0.840 | 0.928 |
| Connectedness to nature | -0.12 | 0.908 | 0.934 | -1.26 | 0.214 | 0.350 | -0.66 | 0.511 | 0.681 |

Note: *q*-values represent FDR-adjusted *p*-values. Significance after correction is denoted as *q* < 0.05.
